# Aerobic Fitness Evaluation during Walking Tests Identifies the Maximal Lactate Steady State

**DOI:** 10.1100/2012/769431

**Published:** 2012-05-01

**Authors:** Guilherme Morais Puga, Eduardo Kokubun, Herbert Gustavo Simões, Fabio Yuzo Nakamura, Carmen Sílvia Grubert Campbell

**Affiliations:** ^1^Catholic University of Brasília, 72022-900 Brasília, DF, Brazil; ^2^Institute of Bioscience, Department of Physical Education, São Paulo State University, 13506-900 Rio Claro, SP, Brazil; ^3^Department of Physical Education, State University of Londrina, 86051-990 Londrina, PR, Brazil

## Abstract

*Objective*. The aim of this study was to verify the possibility of lactate minimum (LM) determination during a walking test and the validity of such LM protocol on predicting the maximal lactate steady-state (MLSS) intensity. *Design*. Eleven healthy subjects (24.2 ± 4.5 yr; 74.3 ± 7.7 kg; 176.9 ± 4.1 cm) performed LM tests on a treadmill, consisting of walking at 5.5 km · h^−1^ and with 20–22% of inclination until voluntary exhaustion to induce metabolic acidosis. After 7 minutes of recovery the participants performed an incremental test starting at 7% incline with increments of 2% at each 3 minutes until exhaustion. A polynomial modeling approach (LMp) and a visual inspection (LMv) were used to identify the LM as the exercise intensity associated to the lowest [bLac] during the test. Participants also underwent to 2–4 constant intensity tests of 30 minutes to determine the MLSS intensity. *Results*. There were no differences among LMv (12.6 ± 1.7%), LMp (13.1 ± 1.5%), and MLSS (13.6 ± 2.1%) and the Bland and Altman plots evidenced acceptable agreement between them. *Conclusion*. It was possible to identify the LM during walking tests with intensity imposed by treadmill inclination, and it seemed to be valid on identifying the exercise intensity associated to the MLSS.

## 1. Introduction

The blood lactate responses during incremental test preceded by a high-intensity exercise exhibit an U-shape pattern. The exercise intensity associated to the minimum blood lactate concentration ([bLac]) during test was first suggested to be the equilibrium point between blood lactate production and removal [1] and has been called lactate minimum (LM) intensity [2]. The LM test has been applied in several exercise modes and conditions [2–7] and shown to be associated to the maximal lactate steady state (MLSS) [3, 4, 7, 8] which is the gold standard among protocols of aerobic fitness evaluation derived from the [bLac] responses to exercise [9, 10].

 Despite the variations on procedures for the [bLac] elevation [7], the length of the recovery period preceding the incremental test [11], and the number of incremental stages, the validity of LM as an index of MLSS was well demonstrated in running, cycling, and swimming [2, 4, 8], both in laboratory and field conditions [2, 5, 6]. However, to our knowledge the LM protocol has not been applied on walking as an exercise mode yet.

 Walking is an exercise mode practiced by most people of any age or aerobic fitness level [12]. It is clear that walking and running are markedly different in terms of ground reaction force, ground contact time, duty factor, and patterns of mechanical energy fluctuations [13, 14]. Also, human walking is always performed with at least one foot in contact with the ground, which leads to a lower bouncing impact compared with running or jogging. During running exercise the eccentric exercise-induced muscle damage can also be larger than during walking, and this damage can lead to greater inflammatory process and muscle injury [14, 15]. Hence, walking produces less risk for musculoskeletal lower extremity injury than running because it is associated with lower reaction forces in low extremities tendons and joints [13, 15–19].

Walking tests have been used and suggested for physical fitness assessment, training prescription, and rehabilitation in different populations, using maximal and submaximal tests and/or exercises [20–22]. Thereby, the ability of indentifying an exercise intensity that evaluates the aerobic fitness using the LM protocol in walking test can be one more important option and also be applied to people that cannot perform maximum running test because of orthopedic limitations or other mechanical or physiological limitations. Thus, the present study analyzed the possibility to determine the aerobic fitness using the LM protocol during walking tests and the validity of LM on predicting the MLSS intensity.

## 2. Methods

### 2.1. Experimental Approach to the Problem

The participants performed an LM protocol adapted from the original protocol of Tegtubur et al. [2] but walking on treadmill rather than running, with the intensity placed on the treadmill inclination rather than on speed. After the LM intensity identification, we investigated the ability of this test protocol to derive the exercise intensity at the MLSS, determined by walking 30 min constant tests until [bLac] steady state was found. It was hypothesized that it is possible to identify both LM and MLSS during walking test and that these intensities are similar and placed in the upper limit of the heavy exercise intensity domain [23].

#### 2.1.1. Subjects

 Eleven healthy and physically active male college students (24.2 ± 4.5 yr, 74.3 ± 7.7 kg of body mass, 176.9 ± 4.1 cm of height, and 24 ± 2 kg·(m^2^)^−1^ of BMI) volunteered to take part in the study after providing informed consent, as approved by the Local Research Ethics Committee.

#### 2.1.2. Procedures

All the subjects were instructed not to exercise and ingest alcohol or caffeine for at least 24 hours before all the tests and to ingest the last meal at least 2 hours before performing each test. The subjects performed one LM test protocol and constant velocity trials to determine the MLSS on the treadmill (Movement Technology-RT300 PRO). All tests were completed over a 3-week period approximately at the same time of day (±1.0 h) and were interspersed with a minimum of 72 hours and a maximum of one-week period. Before each test, subjects completed 8 min of walking as warm-up (5.5 km·h^−1^, 1% incline). The room temperature was kept constant at 25°C with 60% of humidity during all the tests.

 All tests were performed at a walking velocity of 5.5 km·h^−1^, and the intensity increments were placed only by augmenting the treadmill inclination (%). This velocity was chosen from a pilot study, characterizing walking exercise for these subjects. During all the tests the heart rate (HR) was monitored continuously by a wrist monitor (Polar S810, Finland).

#### 2.1.3. Exercise Tests

Lactate minimum (LM) was adapted from the original protocol proposed by Tegtbur et al. [2] to be performed using a walking exercise mode. After warm-up, subjects performed a maximal constant intensity test (5.5 km·h^−1^) with 20% or 22% of treadmill inclination until volitional exhaustion to induce metabolic acidosis. These intensities were chosen based on the subject's self-reported fitness level and attested in the pilot study to be able to increase the [bLac] above the heavy exercise intensity domain (or the anaerobic threshold and MLSS).

After 7 minutes of passive recovery (according to Tegtbur et al. [2] protocol), blood sample was collected for lactate analysis. Thereafter, the subjects initiated an incremental test beginning with 7% with 2% of inclination increments at each 3 min until volitional exhaustion. The initial exercise intensity was chosen to the subject perform around 5–7 stages, with a minimum [bLac] point around the third and fourth stages. Also the increment was chosen to be increasing approximately 1 MET at each stage, based on the pilot study's result. The consecutive stages were interspersed with 1 minute interval for blood sampling. Peak oxygen uptake (VO_2_), Carbon dioxide output (VCO_2_), ventilation per minute (VE), rating of perceived exertion (RPE), HR, and intensity were obtained in the last completed stage.

**Figure 1:**
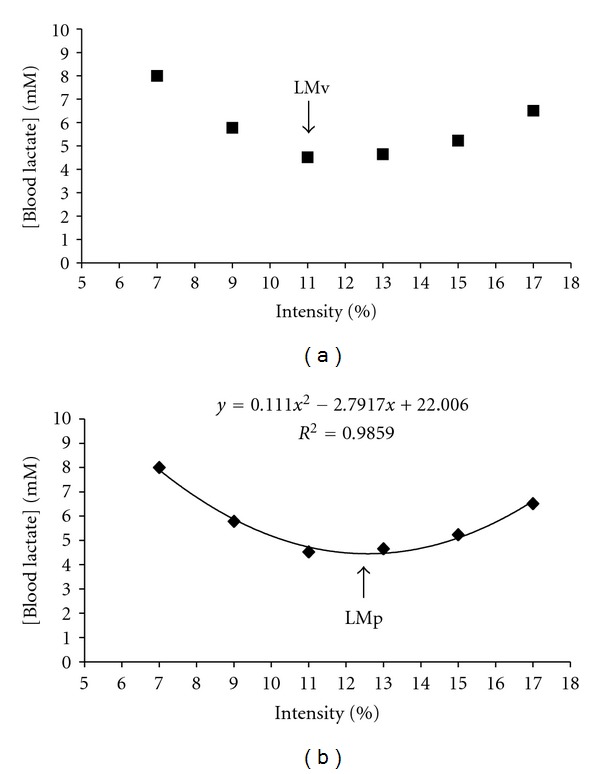
Determination of lactate minimum intensity during LM test using visual inspection (LMv) (a) and polynomial function (LMp) (b).

Identification of lactate minimum intensity (LMi) was identified as the lowest [bLac] during the incremental test by using a visual inspection of the minimum point (LMv) and also by applying a second degree polynomial function (LMp), to allow for the minimum point identification using a mathematical model, as described in Figure 1.

The polynomial function was applied using the Excel program (Microsoft Office), and the LMp was identified from the second-degree derivate of the following equation:

(1) $$\begin{array}{c} \left[\text{La}\right]=a\cdot {\text{intensity}}^{2}+b\cdot \text{intensity}+c,\\ \text{Derivate}\;\text{of}\;\text{the}\;\text{equation}:0\;=2ax+b,\\ x=\underline{-b}\;\text{or}\;\text{LM}\;\text{intensity}\;=\underline{-b},\\ 2.\text{a}\;\;\;\;\;\;\;\;\;\;\;2.\text{a} \end{array}$$

where *a* and *b* are constants and *x* is the intensity corresponding to the parabolic vertices or lactate minimum intensity.

In the maximal lactate steady, state test, the participants performed 30 min constant intensity walking test on treadmill at the LMi. The intensity was increased or decreased by 1% of the treadmill inclination until the MLSS was found. The MLSS was considered the higher intensity that [bLac] increased up to 0.05 mM·min^−1^ between the 10th and 30th minutes [9, 10].

#### 2.1.4. Data Collection Procedures

Respiratory gases measurements: VO_2_, VCO_2_, and VE were measured throughout each test using a Metalyzer 3B (Cortex Biofhysik, Germany) gas analyzer. Expired gases were measured breath by breath and averaged every 10 s. Before each test, the O_2_ and CO_2_ analyses systems were calibrated using ambient air (20.9% O_2_ and 0.03% CO_2_) and standard gases (12.01% O_2_ and 5% CO_2_). The calibration of the turbine flow-meter of the analyzer was performed with a 3 L syringe.

The VO_2_, VCO_2_, VE, and HR results from the LMv intensity were obtained from the mean values of the last 30 s of each stage. The peak intensity variable results were obtained from the last 30 s of the last completed stage. The results from MLSS test were obtained from the mean results between the 10th, 20th, and 30th minutes.

#### 2.1.5. Measurements of Blood Lactate Concentrations

A 25 *μ*L sample of capillary blood was taken from ear lobe and deposited in eppendorf tubes containing 50 *μ*L of NaCl 1%, for blood lactate analysis (YSI 2300 STAT, Yellow Spring Instruments, OH, USA).

#### 2.1.6. Statistical Analysis

Data are presented as mean ± SD. The level of significance was set at *P* < 0.05. One-way ANOVA was used to compare variables corresponding to LMv, LMp, and MLSS, with Tukey as post hoc. When the variables were compared between only the MLSS and LMv, the Student *t*-test were used. The variables' agreements were analyzed by Bland and Altman method [24].

## 3. Results

 All subjects completed the LM tests and it was possible to identify LMv and LMp in all participants (*n* = 11). The mean duration, intensity, and [bLac] corresponding to the metabolic acidosis induction exercise before the incremental test were 283.8 ± 125.0 s, 20.9 ± 1.0%, and 8.5 ± 2.0 mM, respectively.

**Figure 2:**
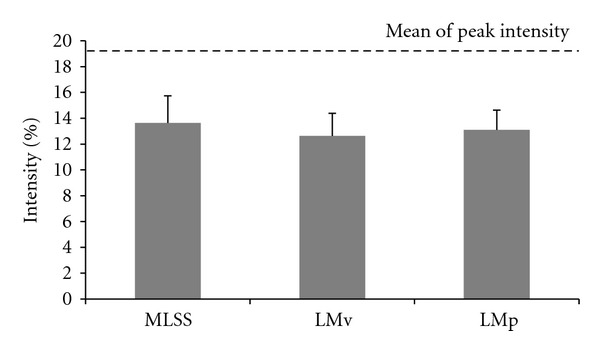
Mean results and standard deviation of intensity (% treadmill inclination) for MLSS, LMv and LMp and the mean peak intensity.

 When the MLSS, LMv, and LMp intensity were compared, the ANOVA showed no difference between their % inclinations (13.6 ± 2.1%, 12.6 ± 1.7%, and 13.1 ± 1.5%, resp.) as displayed in Figure 2.

Table 1 shows that there were no statistical differences between MLSS and LMv regarding blood lactate, VO_2_, HR, or RPE. However, the VCO_2_ and VE, observed in LMv, differed statistically when compared with MLSS.

 The peak VO_2_, VCO_2_, VE, HR, and RPE values at exhaustion in the incremental test were significantly higher (*P* < 0.01) than the ones corresponding to MLSS or LMv.

The MLSS intensity corresponded to 69.2 ± 8.8% of peak intensity and was not different from the relative to peak intensity of LMv (65.7 ± 8.7%).

The same results were found when analyzed the relative to peak values of VO_2_, HR, and RPE between MLSS and LMv, where there was no statistical difference. However, the relative values to peak of VCO_2_ and VE were statistically different.

**Figure 3:**
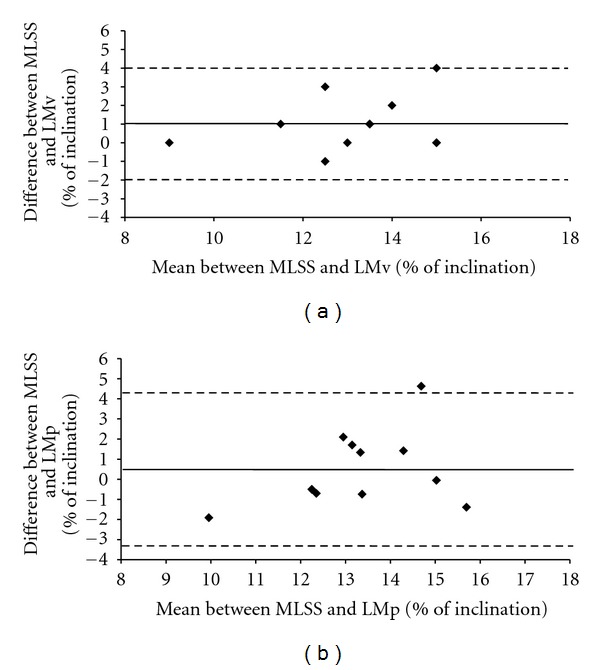
Limits of agreement between MLSS, LMv, and LMp using Bland and Altman method.

The bias ±95% limits of agreement for comparisons between the % inclinations obtained at MLSS and LMv (1.0 ± 2.8%) and at MLSS and LMp (0.5 ± 3.2%) suggest a good agreement between the MLSS and the LM (Figure 3).

**Figure 4:**
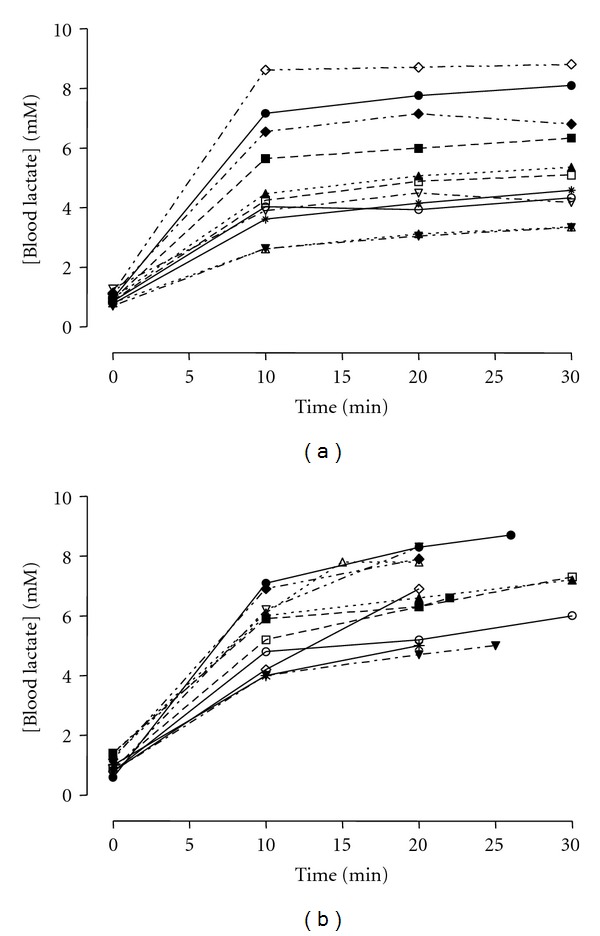
Blood lactate concentrations from all subjects during the 30-min constant test at MLSS intensity (a) and at the intensity 1% above the MLSS (b).

Figure 4 shows the [bLac] from all eleven subjects during the 30 min constant test at the MLSS intensity (Figure 4(a)) and 1% of inclination above the MLSS intensity (Figure 4(b)). At the intensity 1% above the MLSS, only 3 subjects could complete the 30 min without voluntary exhaustion, but none of them had [bLac] steady state according to the criteria used in this study for MLSS determination. It is important to note that at the MLSS intensity the VO_2_ from all participants was also in steady-state, while at the intensity above, there were not steady state pattern (data not shown).

## 4. Discussion

The present study analyzed the possibility to evaluate the aerobic fitness using an LM test protocol during walking and the validity of the LMi to identify the MLSS in walking tests. The main finding was that MLSS intensity determined in walking exercise did not differ from LM intensity both identified by visual inspection and applying polynomial function.

 The exercise intensity corresponding to MLSS is considered a gold standard among the protocols that identify the aerobic fitness using blood lactate responses. According to Beneke et al. [9] the MLSS intensity appears to be affected by the motor pattern of the exercise, and the lactate production and elimination are determined by exercise intensity and mass of the muscles engaged. The steady state of the [bLac] may indicate an overall balance between lactate appearance and disappearance in spite of net lactate production by the primarily engaged muscles. Independently of the exercise mode, the MLSS identification is very important and represents the highest workload that can be maintained over time without a continuous blood lactate accumulation and consequent exercise fatigue [9, 10, 23, 25].

 The results from our study during the 30-min constant trials for MLSS determination are in accordance with those studies that investigated the MLSS in different exercises modes [9, 10, 23]. Figure 4 shows the [bLac] of all participants at MLSS intensity and at the intensity with only 1% of inclination above the MLSS intensity. During the intensity above the MLSS only three participants could complete the 30 min of exercise, but none of them were with [bLac] steady state. These data suggest that all the participants were at an exercise intensity that could not be sustained a long period of time. Based on these results we suggested that the exercise protocol used in our study successfully identified the MLSS intensity in walking test for these participants.

The identification of this intensity could be very important for training prescription aiming to increase maximal and submaximal markers of aerobic capacity [5, 8, 9, 23, 25, 26]. However, it is not practical, because of the number of trials necessary to directly determine this paramount intensity. In this sense, the LM test protocol has shown to be a practical and valid method to predict the MLSS and the anaerobic threshold using a single testing session, since the first study of Tegtbur et al. [2] in the beginning of 1990's until nowadays [3–6, 8, 25, 26].

Many other studies have identified the MLSS and LM in different exercise types, such as running, cycling, rowing, or swimming [5, 8, 25], but to our knowledge this is the first study that has used the identification of this intensity during walking test. The identification of the LM or MLSS in this kind of exercise is very important because walking is the most common exercise modality among population because it has great accessibility [12].

 During walking exercise, loading at the ankle tendons and ligaments is less than during running [13, 16, 19], leading to a lower risk of injuries when compared to running [13, 14, 18]. Then, the protocols using walking tests, like in our study, are interesting to be applied to people that resists to run or among special populations.

 Thus, many authors used and suggested walking tests and exercises for fitness level assessment, rehabilitation, and also training prescription for different populations [12, 20–22] but none of them used this pattern of movement to identify the aerobic fitness by MLSS or LM protocols, which are very useful and important protocols for functional assessment in all kinds of population [8, 23, 26].

 Tegtbur et al. [26] applied the LM protocol in patients with coronary artery disease to investigate if this intensity represents the MLSS intensity, and their results indicated that the LM estimated under lactic acidosis in two successive maximal incremental tests indeed represented the individual MLSS intensity. According to this study, training regimens for these patients could be designed from LM test results. Our results corroborate the use of the LM exercise intensity.

 Our study used 2 consecutive maximal tests during the LM protocol as the study from Tegtbur et al. [26], but it is important to note that our participants were healthy and physically active. The application of this protocol for sedentary and special population could be adapted with two nonmaximal exercise tests in future studies.

During the first exercise used to induce metabolic acidosis, it is important to elevate [bLac] above the equilibrium point between the lactate production and removal around the MLSS. This point can be below the maximal intensity [8, 25], and other kinds of exercises or tests for [bLac] elevation can be used as shown in some studies [5, 7, 25].

 Although some studies have shown that the [bLac] before the incremental test could influence the nadir in LM protocol [27], according to Smith et al. [7] the LM intensity is not dependent upon the lactate elevation levels. Walking at 5·5 km·h^−1^ and with 20–22% inclination was severe enough to induce [bLac] elevation to fulfill the requirements for LM protocol.

Another important consideration is during the incremental test in the LM protocol, which usually is made with six stages, with the LM being identified in the third or fourth stage, and it is not necessary to perform this test until the voluntary exhaustion. In our study, we asked for the participants to perform until the voluntary exhaustion, to be able to identify the maximal parameters (VO_2_, VCO_2_, VE, RER, HR, RPE) during walking tests and compare these values with the results found at the MLSS and LM.

 The [bLac] was not different between MLSS and LM, suggesting that even with hyperlactatemia induction before the incremental tests, these intensities appear to reflect similar [bLac] levels. But according to MacIntosh et al. [8] the absence of any relationship between MLSS intensity and plasma lactate concentration indicates that absolute lactate values comparing different test protocols are probably not relevant to this test.

 Similar to the intensity and [bLac], there was no statistical difference in the VO_2_ results between MLSS and LM, which can probably be explained by the fact that the aerobic energy to support the exercise at these intensities was similar, even with different test protocols or exercise durations, and with or without previous lactic acidosis induction.

 According to our results the LM and MLSS are placed in the upper limit of the heavy exercise intensity domain, and the exercise at these intensities can be performed with [bLac] steady state, and for a long period of time [23]. The results from both absolute and relative LM and MLSS intensities compared with the peak results in the incremental test also support this hypothesis.

 Tables 1 and 2 showed that the VCO_2_ and VE were significantly lower at the LM compared with the MLSS in both relative and absolute values. The lower VCO_2_ may be explained due to the hyperventilation during the 7 min of recovery, induced by the high-intensity exercise before the incremental test [28–30]. The hyperventilation has probably induced a ventilatory alkalosis and consequently produced a lower PaCO_2_ at the beginning of the incremental test [28, 29].

 Furthermore, during the MLSS the exercise was performed longer at an exercise intensity that produces a higher acidosis buffering and consequently a higher VCO_2_ [29, 30]. These VCO_2_ responses also explain the lower RER (data not shown) and VE, which are consequence and are in accordance with the VCO_2_ response.

## 5. Conclusion

Thus, these results suggested that it is possible to identify the LM intensity during walking tests with intensity imposed by treadmill inclination for aerobic fitness evaluation. Moreover the LM protocol appears to be a valid method to identify an exercise intensity that can be sustained at the MLSS.

The LM protocol had been shown to be a valid test for MLSS determination using a single exercise test session, and this intensity can be used for aerobic capacity and fitness level evaluation, in all populations. Besides the assessment importance of the LM, it is very important for training prescription based on this parameter, for aerobic capacity improvement. The protocol proposed by our study is one more important option to be applied in people that are not able to perform maximal running tests or tests with high treadmill velocities, such as sedentary, obese, diabetics, and people that cannot perform the running movement pattern.

## Figures and Tables

**Table 1:** Results corresponding to maximal lactate steady-state intensity (MLSS), lactate minimum identified by visual inspection (LMv) and at the peak intensity. Intensity; [bLac] blood lactate concentration; VO_2_: oxygen uptake; VCO_2_: carbon dioxide output; VE: minute ventilation; HR: heart rate; RPE: rate of perceived exertion.

	Intensity	[bLac]	VO_2_	VCO_2_	VE	HR	RPE
	(%)	(mM)	(L·min^−1^)	(L·min^−1^)	(L·min^−1^)	(bpm)	
MLSS	13.6 ± 2.1	5.1 ± 1.8	2.8 ± 0.4	2.8 ± 0.5	85.8 ± 12.1	170 ± 8	16 ± 2
LMv	12.6 ± 1.7	4.7 ± 1.7	2.8 ± 0.4	2.4 ± 0.6*	75.7 ± 15.8*	167 ± 12	15 ± 2
Peak	19.5 ± 2.4^#^	—	3.3 ± 0.5^#^	3.5 ± 0.5^#^	126.2 ± 22.2^#^	187 ± 8^#^	20 ± 0^#^

*P < 0.05 in relation to MLSS.

^#^ *P* < 0.01 in relation to MLSS and LMv.

**Table 2:** General results corresponding to maximal lactate steady-state intensity (MLSS), lactate minimum, relative to peak variables. Intensity; VO_2_: oxygen uptake; VCO_2_: carbon dioxide output; VE: minute ventilation; HR: heart rate; RPE: rate of perceived exertion.

	Intensity	VO_2_	VCO_2_	VE	HR	RPE
	% of peak	% of peak	% of peak	% of peak	% of peak	% of peak
MLSS	69.2 ± 8.8	87.0 ± 8.4	80.5 ± 9.3	68.7 ± 7.3	91.0 ± 2.9	78.8 ± 7.8
LMv	65.7 ± 8.7	80.4 ± 12.4	70.1 ± 13.0*	60.7 ± 11.2*	89.4 ± 3.5	75.0 ± 7.1

*P > 0.05 in relation to MLSS.
